# Respiratory Sinus Arrhythmia as an Index of Vagal Activity during Stress in Infants: Respiratory Influences and Their Control

**DOI:** 10.1371/journal.pone.0052729

**Published:** 2012-12-26

**Authors:** Thomas Ritz, Michelle Bosquet Enlow, Stefan M. Schulz, Robert Kitts, John Staudenmayer, Rosalind J. Wright

**Affiliations:** 1 Department of Psychology, Southern Methodist University, Dallas, Texas, United States of America; 2 Department of Psychiatry, Boston Children's Hospital, Boston, Massachusetts, United States of America; 3 Department of Psychiatry, Harvard Medical School, Boston, Massachusetts, United States of America; 4 Department of Psychology, University of Würzburg, Würzburg, Germany; 5 Comprehensive Heart Failure Center (CHFC), University of Würzburg, Würzburg, Germany; 6 Department of Mathematics and Statistics, University of Massachusetts, Amherst, Massachusetts, United States of America; 7 Channing Laboratory, Department of Medicine, Brigham & Women's Hospital, Harvard Medical School, Boston, Massachusetts, United States of America; 8 Department of Environmental Health, Harvard School of Public Health, Boston, Massachusetts, United States of America; University of Houston, United States of America

## Abstract

Respiratory sinus arrhythmia (RSA) is related to cardiac vagal outflow and the respiratory pattern. Prior infant studies have not systematically examined respiration rate and tidal volume influences on infant RSA or the extent to which infants' breathing is too fast to extract a valid RSA. We therefore monitored cardiac activity, respiration, and physical activity in 23 six-month old infants during a standardized laboratory stressor protocol. On average, 12.6% (range 0–58.2%) of analyzed breaths were too short for RSA extraction. Higher respiration rate was associated with lower RSA amplitude in most infants, and lower tidal volume was associated with lower RSA amplitude in some infants. RSA amplitude corrected for respiration rate and tidal volume influences showed theoretically expected strong reductions during stress, whereas performance of uncorrected RSA was less consistent. We conclude that stress-induced changes of peak-valley RSA and effects of variations in breathing patterns on RSA can be determined for a representative percentage of infant breaths. As expected, breathing substantially affects infant RSA and needs to be considered in studies of infant psychophysiology.

## Introduction

The respiratory system modulates autonomic outflow to various organ sites, including the heart, trachea, bronchi, and blood vessels [1], [2]. Respiratory sinus arrhythmia (RSA), or its frequency-domain equivalent, high-frequency heart rate variability, is often used as an index of cardiac vagal activity [3]–[6]. The peak-valley (or peak-to-trough) method provides a common time-domain index of RSA [4], [5] which is extracted breath-by-breath by subtracting the minimum heart rate (HR) during expiration from the maximum HR during inspiration; or, alternatively, it can be calculated with the cardiac inter-beat interval (IBI), i.e., minimum IBI during inspiration subtracted from maximum IBI during expiration. Compared to frequency domain analyses, the peak-valley method has the advantage of providing an RSA index that allows for a breath-by-breath analysis and thus can be extracted for very short time frames. Frequency-domain measures of high-frequency heart rate variability, on the other hand, require a minimum of at least two minutes of uninterrupted recordings to generate stable estimates [3]. Nevertheless, under adequate conditions the two methods are often highly correlated [7].

In older children and adults, these respiration-related fluctuations have found broad application in research on autonomic function (e.g., [8], [9]), emotion and stress (e.g., [10]–[13]), psychopathology (e.g., [14]–[18]), and clinical cardiovascular research (e.g., [19], [20]). Research in adults has also shown that the amplitude of RSA decreases with respiration rate (f_R_) and increases with tidal volume (V_T_) independent from changes in cardiac vagal activity (e.g., [5], [9], [21]–[25]). Systematic variations in f_R_ and V_T_ can explain up to 60% of the variance in RSA [23], [26]. A respiratory gating model has been proposed that considers an influence of both central respiratory drive and peripheral lung inflation on membrane potentials of preganglionic vagal motor neurons in the nucleus ambiguous [1], [27]. Rhythmic hyperpolarizations and depolarizations with inspiration and expiration, respectively, constrain the output of these neurons so that their maximum output occurs in the post-inspiratory phase [28]. The background firing rate of these neurons is determined by various inputs (e.g., baroreceptors, chemoreceptors, laryngeal neurons, trigeminal afferents), and thus may vary independently from its modulation by the respiratory gating mechanism. Thus, the same amount of vagal output per time unit can result from slow versus fast respiration rates, which are associated with more or less pronounced gating of vagal output, respectively [5]. Under less pronounced gating conditions, vagal outflow is less dampened during inspiration and less enhanced during expiration, whereas under more pronounced gating conditions there is stronger dampening in inspiration and stronger enhancement in expiration. Overall, the same net-output of vagal activation reaches the heart per time unit, although RSA would be drastically different between both conditions. Notably, there is inconsistency in the literature about the role peripheral lung inflation plays in the respiratory gate [3], [29], [30]. Strong arguments have also been raised for central gating mechanisms [29] versus peripheral baroreflex mechanisms [30] as contributors to RSA.

Respiratory modulation of cardiac activity has also been described in the first year of life, although with mixed success (e.g., [31]–[41]). In addition, a growing number of studies have explored the susceptibility of infant RSA to environmental stimulation or psychological challenges (e.g., [6], [42]–[45]), but success has also been variable [46]–[49]. None of these studies have considered potential influences of respiration on RSA amplitude, which may have affected the power to explore the extent to which stress impacts RSA in this population. The need to explore methods to correct RSA for respiratory influences has been underscored as an important area for future research guidelines on RSA measurement and interpretation [3], but so far little has been published in this area. Specifically, to our knowledge, there are no published studies in infants examining respiratory correction procedures for RSA. In adults, the normalization of RSA by V_T_ (or the transfer function of RSA per mL V_T_) has been considered by some as a useful approach to control for respiratory pattern influences [8], [9], [23], [25]. It capitalizes on a usually tight inverse correlation between V_T_ and respiration rate (f_R_), which maintains adequate levels of gas exchange. RSA/V_T_ has been used in ambulatory measurements to control for respiratory effects when influences of metabolic changes on heart rate (HR) can be considerable. Under these circumstances, f_R_ and V_T_ are usually highly correlated and additional adjustments for f_R_ may not be necessary after accounting for V_T_ effects [50]. However, during laboratory assessments with minimal change in metabolic demands and emotional challenge, V_T_ and f_R_ effects may be more dissociated and adjustment for both would be more optimal [26].

In addition, sensitivity of detecting RSA changes may be affected by the fact that infant autonomic regulation may not consistently provide the conditions to observe a valid RSA. Infants might breathe at frequencies that do not allow an extraction of RSA, i.e. when f_R_>HR/2 [5], [41], [52]–[53]. These short breaths violate the Nyquist rate criterion, which requires the sampling rate to be at least twice as high as the frequency of interest. The extent to which this occurs and its dependency on challenging experimental conditions, which may be associated with increases in f_R_ is not well characterized. It may, however, put constraints on the ability to detect a regular rhythm of acceleration and deceleration of HR with inspiration and expiration and therefore not provide a sensitive estimator of vagal outflow to the heart. Frequency-domain analysis of HR variability assumes an extent of consistency in the cardiorespiratory coupling that may not be realistic for the first few months of life. Because both the respiratory center and the vagal system undergo maturation during this period [54]–[56], the extent to which f_R_ is too fast may be an indicator of the immaturity of cardiorespiratory integration. Breath-by-breath time-domain analysis of RSA allows an analysis of the frequency of breaths that adhere to basic peak-valley RSA criteria requiring at least two IBIs within the time window constituted by one breath, with the shorter IBI that indicates faster HR preceding the longer IBI that indicates slower HR.

We sought to apply correction for within-individual effects of f_R_ and V_T_ on RSA, as done in research on adults [5], [26], [51], to infant data in order to examine whether similar procedures provide well-suited tools for addressing respiratory influences on RSA in this population. The aims of this study were as follows: (i) We sought to explore whether sufficient regularity existed in the cardio-respiratory interaction in 6-month old infants across laboratory conditions of alert play and psychosocial stress to allow for an extraction of RSA. We focused particularly on the occurrence of breaths that would be too short to allow a valid determination of RSA and thus might reduce the extent to which a rhythmic cardiorespiratory coupling could be observed. (ii) We sought to characterize the extent to which variation in the respiratory pattern (changes in f_R_ and V_T_) influences RSA amplitude in infants. (iii) We also sought to explore whether removal of RSA variance attributed to the respiratory pattern in infants could improve the demonstration of vagal withdrawal during psychosocial stress. With regard to the employed psychosocial stressor, we expected a reduction in respiratory pattern-corrected RSA because parasympathetic withdrawal is often part of a general response to behavioral challenges and distress. Stimulation of the hypothalamic defense area of animals has been shown to reduce excitatory input into preganglionic cardiac vagal motor neurons via the periaqueductal gray [57], [58]. Similarly, whereas orienting towards a novel stimulus has been associated with cardiac vagal excitation, stressful laboratory stimulation such as mental arithmetic challenge [59]–[62] or chronic life-stress [63], has been linked to dampening of baroreflex activity. A better demonstration of this effect would speak to the validity of respiration-corrected versus uncorrected RSA parameters in studies of infants.

## Methods

### Participants

Mother-infant pairs (*N* = 23) were recruited from the general population through obstetrics and gynecology clinics in Boston, MA. Mothers were ≥18 years old and had a singleton gestation. Additional exclusion criteria for the current protocol included maternal report during pregnancy of alcohol consumption exceeding seven drinks/week prior to pregnancy recognition or any alcohol consumption or smoking 10 or more cigarettes/day following pregnancy recognition [64]. Infants at high risk of neurodevelopmental disorders were also excluded (gestational age <32 weeks; birth weight <5.5 lbs; congenital abnormalities; neurological injury). Procedures were approved by Institutional Review Boards at participating institutions; mothers provided written informed consent.

### Physiological and observational measurements

Respiration and cardiac activity were measured using the LifeShirt System (VivoMetrics, Inc., Ventura, CA), a non-invasive ambulatory respiratory inductance plethysmography device [65] adapted to infants. A sleeveless Velcro shirt with built-in inductance bands and cables for a 3-lead ECG was fitted to the infant, with electrodes attached to the sternum, the lower left rib, and the left clavicle. Raw signals from the two inductance bands and the ECG leads were amplified, A/D converted and stored continuously either in an attached PDA-size recorder or in real time on an IBM compatible laptop. For volume calibration of the inductance band output, the Qualitative Diagnostic Calibration procedure [66] was applied.

Videotaped sessions were scored second-by-second by three trained coders to rate infant activity level throughout the protocol. Disagreements were conferenced until consensus was reached. Infant activity level was rated on a 4-point scale: 0 = quiet motor, 1 = slow/mild, 2 = moderate, 3 = pronounced [modified from 47]. Forty-eight percent of tapes were coded for inter-rater reliability (*ICC* = .92). The percentage of time an infant spent in each activity level was calculated, multiplied by the activity level value, and summed, resulting in a range from 0 (maximum of “quiet motor”) to 300 (maximum of “pronounced”).

### Behavioral challenge: The Still-Face Paradigm

**Figure 1:**
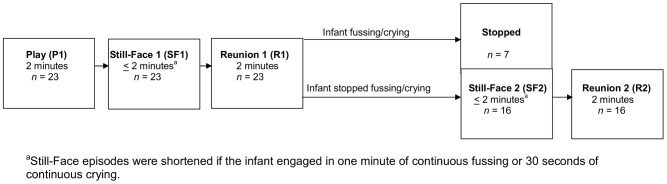
Overview of the Still-Face Paradigm.

Following acclimatization to the recording system, infants were placed in a standard baby car seat mounted on a table 3 feet across from their mother seated at infant eye level. The mother then interacted with the child for 3 minutes either reading a story or blowing bubbles. Subsequently, the Still-Face Paradigm (SFP) was administered (Figure 1). This paradigm is an established protocol for assessing infants' responses to brief, moderate social stress [45] involving three 2-minute episodes: (i) Play: mothers play with their infant, (ii) Still-Face: mothers maintain a neutral facial expression, avoid touching or vocalization, and (iii) Reunion: recovery, mothers resume playing with their infant. If an infant showed 1 minute of continuous fussing or 30 seconds of continuous crying, the still-face period was terminated and the reunion period was administrated next. A second still-face and reunion were administered to introduce a more sustained level of challenge [67]. Additional episodes were not administered when infants did not stop crying or fussing during the first reunion.

### Data reduction and analysis

Breath-by-breath analysis of timing, volume, and flow parameters of respiration and HR was done using ViovoLogic software. Breath-by breath total respiratory cycle time (T_TOT_), V_T_, and interbeat-intervals (IBI) of the ECG were extracted. IBI artifacts were edited by raters with extensive training. Editing was always double checked by a second rater and any discrepancies were resolved by discussion and additional expert input if necessary. Peak-valley analysis of RSA [23] was obtained from customized software [68] providing additional statistics on the breath-by-breath frequency of RSA. Because of the possibility that RSA was phase-advanced relative to the breathing cycle [4], IBIs that started before the onset of inspiration and overlapped with it were included in the analysis. Percentages of breaths were calculated in which RSA was zero due to violation of the Nyquist criterion (T_TOT_<IBI_t1_+IBI_t2_, or HR/2>f_R_, where t1 is the first IBI associated with the onset of inspiration and t2 is the subsequent IBI), as well as breaths that did not adhere to the basic peak-valley criterion of IBI_minimum_ preceding IBI_maximum_. In addition, peak-valley RSA normalized by V_T_ (logRSA/V_T_) and adjusted for T_TOT_, which has been shown to be an improved estimator of cardiac vagal activity in adults [5], [9], [25], was calculated by within-regression analysis for each subject. Log-transformation was applied to improve distributional characteristics. The grand mean of unadjusted logRSA/V_T_ was added to the residual to obtain the respiration-corrected logRSA/V_Tc_

One-way repeated measures ANOVAs explored systematic variations of RSA T_TOT_, V_T_ and HR across the SFP. Primary analysis included 16 infants who participated in all five episodes; supplementary analysis was performed for all 23 infants who completed the first three episodes. Greenhouse-Geisser correction was applied where appropriate and results are reported with original *df*s and corrected p-levels. Within-individual associations of RSA with respiration were explored using hierarchical multiple linear regressions, with T_TOT_ entered in Step 1 and V_T_ in Step 2. Performance of logRSA/V_Tc_, (subscript “c” denotes adjustment for T_TOT_ within infant) was then compared to the uncorrected peak-valley RSA. Supplementary analyses compared an additional range of respiration-corrected and uncorrected RSA indices: RSA_c_, RSA/V_T_, logRSA = logarithm(RSA+1), logRSA_c_, and logRSA/V_T_ = logarithm(RSA/V_T_)+1. For corrected indices, the overall mean of each uncorrected RSA parameter (for *n* = 23 or *n* = 16, respectively) was added to the respective residual to obtain the absolute values of the respiration-corrected RSA parameter. All breaths that allowed for an RSA extraction were included in these analyses, as well as those with no detectable peak-valley RSA, which were scored as zero. Breaths that were too fast for RSA extraction were dropped from the analyses. Multiple post-hoc comparisons of means were performed by Bonferroni-adjusted *t-*tests (overall *p*<.05).

We also used linear mixed models [69] to explore the potential role of physical activity as a time-dependent covariate of RSA changes. Overall, we expected reductions in RSA in Still-Face 1 and 2 relative to Play. We expected these differences to be observed most clearly for respiration-corrected indices of RSA (particularly with logRSA/V_Tc_, which is corrected for both, V_T_ and T_TOT_) and would be independent of physical activity.

## Results

### Sample characteristics

The sample characteristics have been previously described in detail [64]. Briefly, infants were full-term (gestational age *M* = 39.0 weeks, *SD* = 1.7 weeks) and of normal birth weight (*M* = 3431.5 grams, *SD* = 538.7 grams), primarily minorities (35% Black, 30% White, 26% Multi-racial, and 9% Hispanic), and 48% were male. Assessments occurred when infants were approximately 6.5 months old (*M* = 27.6 weeks, *SD* = 1.4 weeks). Sixteen infants completed both Still-Face test sequences; an additional 7 infants were too distressed during Reunion 1 to continue. There were no significant differences between infants who did and did not complete the full protocol on sex, race/ethnicity, age at assessment, gestational age, or birth weight.

### RSA frequency across episodes

When considering individual SFP episodes, between 5.9 and 12.6% of breaths (on average) were too short to accommodate more than one full IBI for RSA analysis, although in individual infants and experimental phases, up to 58.6% of the breaths were too short (Table 1). Demographic or post-partum variables were not systematically associated with the percentage of breaths that were too short. Among the breaths that were long enough to allow RSA extraction, 41% showed a valid RSA, with the remaining not adhering to the peak-valley criterion of IBI_minimum_ preceding IBI_maximum_.

**Table 1:** Percentages of breaths too short to allow extraction of two inter-beat intervals for peak-valley RSA calculation in infants.

	Play	Still-Face1	Reunion1	Still-Face2	Reunion2	Time (n = 23)		Time (n = 16)	
	(*n* = 23)	(*n* = 23)	(*n* = 23)	(*n* = 17)	(*n* = 16)	*F*	*ηp^2^*	*F*	*ηp^2^*
						2.59 †	.105	1.87	.111
Mean	9.0	5.9	10.3	10.9	12.6				
Range	0.8–42.3	0–33.3	0–28.7	0–44.0	0–58.2				

*Note:* RSA = respiratory sinus arrhythmia; Frequencies across episodes (as % of analyzed breaths) and ANOVA time effects for infants with one Still-Face Test (*n* = 23, *df* = 2,44) and two Still-Face Tests (*n* = 16, *df* = 4,60).

† *p*<.10.

### Variation of peak-valley RSA amplitude, respiration, and heart rate across still-face episodes

**Figure 2:**
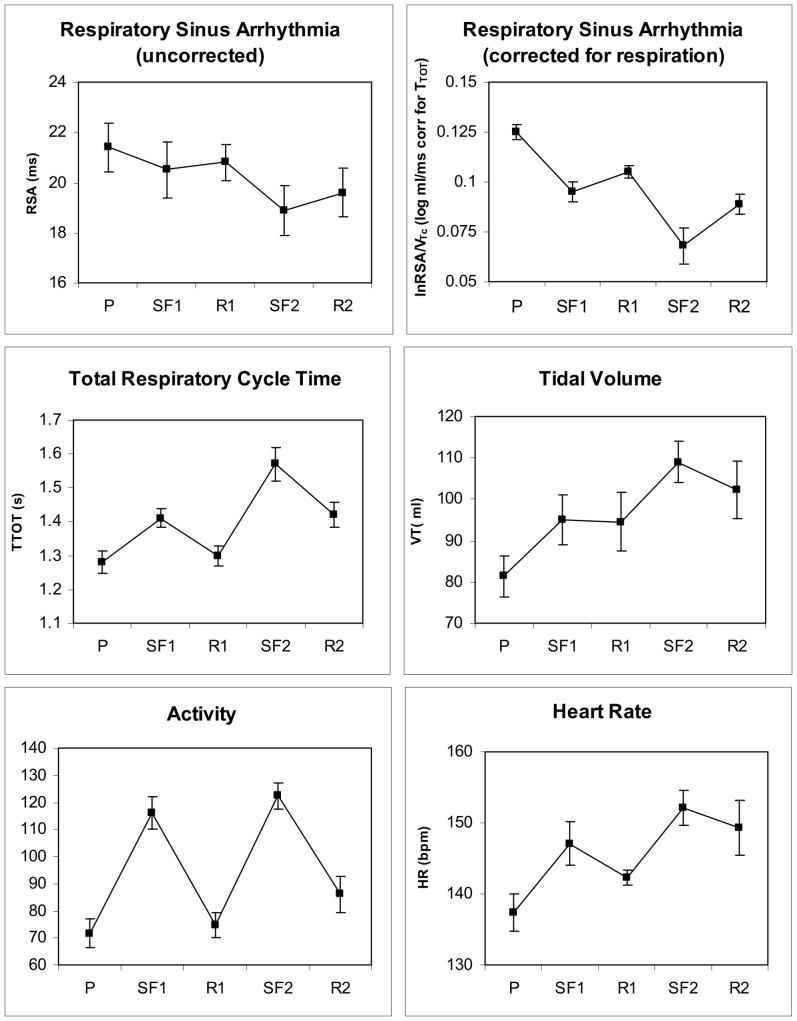
RSA (uncorrected and corrected for respiration), respiratory parameters, physical activity and heart rate across the two Still-Face Test challenges (n = 16).

Uncorrected peak-valley RSA did not significantly vary across the first still face challenge for the complete sample of infants (*n* = 23), but it was significant for the subsample that completed both episodes (*n* = 16; Table 2 and 3; Figure 2, upper left panel).

**Table 2:** Means ± standard deviations of respiration-uncorrected and respiration-corrected RSA indices, respiration measures, and HR for Still-Face Test episodes.

	Play	Still-Face1	Reunion1	Still-Face2	Reunion2
Test 1 (*n* = 23)					
RSA (ms)	11.4±4.1	9.8±4.8	10.8±5.9		
logRSA (log ms)	1.47±0.22	1.26±0.29	1.35±0.29		
RSA_c_ (ms)	12.0±1.8	10.0±1.7	11.3±3.1		
logRSA_c_ (log ms)	1.48±0.12	1.29±0.17	1.38±0.21		
RSA/V_T_ (ms/mL)	0.168±0.068	0.126±0.066	0.129±0.057		
logRSA/V_T_ (log ms/mL)	0.136±0.048	0.102±0.048	0.107±0.042		
RSA/V_Tc_ (ms/mL)	0.172±0.028	0.134±0.033	0.136±0.026		
logRSA/V_Tc_ (log s/mL)	0.139±0.020	0.108±0.022	0.112±0.020		
T_TOT_ (s)	1.31±0.17	1.43±0.14	1.34±0.16		
V_T_ (mL)	86.7±27.4	103.1±24.4	105.3±36.1		
HR (b/min)	135.0±11.5	146.6±12.6	144.5±11.2		
Test 1 and 2 (*n* = 16)					
RSA (ms)	10.6±3.9	9.1±4.5	10.0±3.0	7.5±4.1	9.1±3.8
logRSA (log ms)	1.43±0.20	1.22±0.29	1.39±0.29	1.08±0.32	1.28±0.31
RSA_c_ (ms)	10.5±1.4	8.8±1.5	10.1±1.32	6.6±2.7	8.7±2.5
logRSA_c_ (log ms)	1.40±0.12	1.20±0.17	1.37±0.16	1.03±0.27	1.24±0.23
RSA/V_T_ (ms/mL)	0.160±0.058	0.120±0.061	0.130±0.047	0.086±0.061	0.110±0.052
logRSA/V_T_ (log ms/mL)	0.132±0.042	0.100±0.046	0.109±0.035	0.072±0.045	0.093±0.041
RSA/V_Tc_ (ms/mL)	0.153±0.029	0.115±0.030	0.124±0.022	0.080±0.051	0.105±0.044
logRSA/V_Tc_ (log ms/mL)	0.125±0.021	0.095±0.022	0.105±0.016	0.068±0.037	0.089±0.032
T_TOT_ (s)	1.28±0.14	1.41±0.11	1.30±0.12	1.57±0.20	1.42±0.15
V_T_ (mL)	81.4±19.6	94.9±22.6	94.5±26.7	108.9±21.5	102.3±26.4
HR (b/min)	137.4±10.5	147.1±12.5	142.3±10.2	152.1±15.5	149.3±16.4

*Note:* HR = heart rate; V_T_ = tidal volume; RSA = respiratory sinus arrhythmia; T_TOT_ = total respiratory cycle time; HR = heart rate; RSA/V_T_ = RSA normalized by V_T_; c = adjusted for T_TOT_; logRSA = logarithm(RSA+1); logRSA/V_T_ = logarithm(RSA/V_T_)+1.

**Table 3:** Overall ANOVA time effects (df = 4,66 or 2,44) for changes in physiological parameters across Still-Face Tests and paired t-tests (df = 15 or 22) testing changes from Play to Still-Face episodes 1 and 2.

	Overall ANOVA effect			P vs. SF1		P vs. SF2	
	*F*	*p*	*η_p_^2^*	*T*	*p*	*t*	*p*
Test 1 (*n* = 23)							
RSA (mL)	1.69	.197	.071	2.75	.012		
logRSA/V_T_c (log ms/mL)	13.43	.001	.379	4.99	.001		
T_TOT_ (s)	4.54	.019	.171	2.56	.018		
V_T_ (mL)	8.53	.001	.279	4.02	.001		
HR (b/min)	16.16	.001	.423	7.04	.001		
Test 1 and 2 (*n* = 16)†							
RSA (mL)	4.36	.004	.225	3.36	.008	3.75	.004
logRSA/V_T_c (log ms/mL)	8.23	.001	.354	4.74	.001	4.51	.001
T_TOT_ (s)	12.50	.001	.454	3.13	.014	7.33	.001
V_T_ (mL)	7.09	.001	.321	3.30	.010	5.46	.001
HR (b/min)	7.74	.001	.340	4.95	.001	4.83	.001

*Note*: P = Play episode; SF1 = Still-Face episode 1; SF2 = Still-Face episode 2; T_TOT_ = total respiratory cycle time; V_T_ = tidal volume; RSA = respiratory sinus arrhythmia; logRSA/V_T_c = logarithm of RSA normalized by V_T_ (logarithm(RSA/V_T_)+1), adjusted for T_TOT_; HR = heart rate.

† *p*-level of both *t*-tests for each index are Bonferroni-adjusted in this subgroup.

T_TOT_, V_T_, and HR all increased from Play to Still-Face episodes for the full sample and for the subsample that completed both still-face challenges (Table 2 and 3), indicating slower and deeper breathing paired with tachycardia. Figure 2 shows means of respiratory parameters and HR of the latter subsample. During Reunion episodes, physiological parameters did not fully return to levels of the initial Play episode.

### Association of RSA amplitude with respiratory parameters

In one-half to two-thirds of infants, RSA was significantly or marginally higher with longer T_TOT_ as shown by positive associations between the two parameters (Table 4). Moreover, in 20 of the 23 infants RSA showed significant or marginal (*p*<.10) associations with at least one of the respiratory parameters, T_TOT_ or V_T_. Across infants, 1.4 to 2.3% of the within-individual RSA variance was explained by T_TOT_, depending on whether TT_OT_ or V_T_ was entered first into the hierarchical multiple regression model - for individual infants this could be as high as 10.9%. V_T_ contributed additional systematic variance to RSA, but only in one-third to one-half of the infants (depending on the order that predictors were entered into the equation). Across infants 0.4 to 0.8%, variance was explained, for V_T_ entered first or second, respectively, into the model, and for individual infants this could reach a maximum of 3.9% of the variance explained. The overall regression models were significant in eight cases and showed a trend (*p*<.10) towards significance in another two cases.

**Table 4:** Within-individual association of infant RSA with respiratory parameters T_TOT_ entered in Step 1 and V_T_ entered in Step 2, or in reverse order, calculated across all episodes of the Still-Face Paradigm.

	T_TOT_ (s)			V_T_ (mL)			Multiple *R*	
	*mdn* Δ*R* ^2^	range Δ*R* ^2^	freq. *p*<.10†	*mdn* Δ*R* ^2^	range Δ*R* ^2^	freq. *p*<.10†	*mdn*	range
Entered as Step 1	.014§	.000–.095	12/23	.004	.000–.037	8/23	.179	.055–.364
Entered as Step 2	.023	.001–.109	17/23	.008	.000–.039	12/23		

*Note:* T_TOT_ = total respiratory cycle time; V_T_ = tidal volume; RSA = respiratory sinus arrhythmia.

† frequency of positive within-individual correlations for which *p*<.10.

§ range of *n* = 126–444 breaths.

### RSA amplitude controlled for respiration: Response to still-face challenge

**Figure 3:**
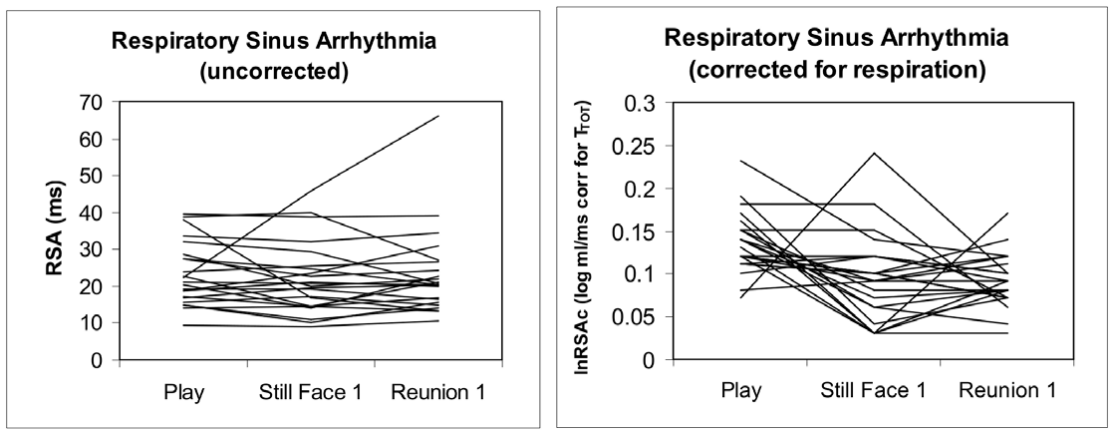
Individual trajectories of RSA uncorrected and corrected for respiration (both tidal volume and respiration rate) across the three episodes of the first Still-Face Test (n = 23).

The respiration-corrected index of RSA, logRSA/V_T_c, showed significant variation across Still-Face episodes (Table 2 and 3), with lower values during Still-Face versus Play episodes. Thus, the corrected index was superior over the uncorrected index by yielding significant changes in the expected direction and explaining more variance across episodes (Table 3; note that time effects in Table 3 refer to the amplitude of RSA whereas time effects in Table 1 refer to percentages of breaths that were too short for RSA extraction). Figure 3 demonstrates the more uniform response profiles with corrected RSA showing reductions from Play to Still-Face episodes. In general, log-transformed and respiration corrected RSA indices showed effects on RSA at a higher significance level than uncorrected indices.

Further exploration of all indices that corrected for respiration in some manner (normalization by V_T_ and/or adjusted for T_TOT_) consistently showed significant variation across SFP episodes (Table 5), with lower values during still-face vs. play episodes. LogRSA/V_T_ performed best among these additional indices, reaching the level of variance explained by logRSA/V_T_c. Corrected indices performed equally well in the total sample completing the first Still-Face test and the subsample completing both tests.

**Table 5:** ANOVA time effects (*df* = 4,60 or 2,44) and paired *t*-tests (*df* = 15 or 22) measuring reduction in additional respiration-uncorrected and corrected RSA indices during Still-Face Test 1 and 2.

	ANOVA time effect			P vs. SF1		P vs. SF2	
	*F*	*p*	*η_p_^2^*	*t*	*p*	*t*	*p*
Test 1 (*n* = 23)							
logRSA (log ms)	6.06	.009	.216	4.84	.001		
RSAc (ms)	3.52	.058	.138	4.12	.001		
logRSAc (log ms)	5.95	.008	.213	4.52	.001		
RSA/V_T_ (ms/mL)	11.75	.001	.348	4.26	.001		
logRSA/V_T_ (log ms/mL)	14.14	.001	.391	5.18	.001		
RSA/V_T_c (ms/mL)	11.44	.001	.342	4.15	.001		
Test 1 and 2 (*n* = 16)							
logRSA (log ms)	6.76	.003	.311	3.90	.001	4.90	.001
RSAc (ms)	7.93	.002	.346	4.09	.001	4.69	.001
logRSAc (log ms)	7.62	.002	.337	3.77	.004	5.05	.001
RSA/V_T_ (ms/mL)	6.74	.002	.310	4.72	.001	4.06	.001
logRSA/V_T_ (log ms/mL)	8.13	.001	.352	4.98	.001	4.54	.001
RSA/V_T_c (ms/mL)	6.87	.002	.315	4.53	.001	4.06	.001

*Note:* V_T_ = tidal volume; RSA = respiratory sinus arrhythmia; T_TOT_ = total respiratory cycle time; HR = heart rate; RSA/V_T_ = RSA normalized by V_T_; c = adjusted for total respiratory cycle time; logRSA = logarithm(RSA+1); logRSA/V_T_ = logarithm(RSA/V_T_)+1.

*p*-level of both tests Bonferroni-adjusted for all indices.

### The role of physical activity in RSA change

Infants' physical activity increased substantially from Play to Still-Face and decreased during Reunion episodes (Figure 2, left bottom panel). ANOVA time effects were significant for both the full sample, *F*(2,44) = 43.53, *p*<.001, *ε* = .81, *η_p_^2^* = .664, and the subsample, *F*(4,56) = 20.26, *p*<.001, *ε* = .65, *η_p_^2^* = .591. Using activity as a time-varying covariate in the LMM analysis of uncorrected RSA did not result in significant RSA variation across episodes. Physical activity was a significant covariate for the subset of infants undergoing both tests. However, logRSA/V_T_c continued to vary significantly across episodes when residualized for physical activity, and activity was not a significant covariate (Table 6). Similarly, T_TOT_, V_T_, and HR continued to vary significantly. In these analyses, the physical activity covariate was only significant for HR.

**Table 6:** RSA, respiration, and heart rate across Still-Face Test episodes controlled for physical activity: Linear mixed model overall *F* tests (*df* = 2 or 4, 50.0 to 67.7), time-varying covariate activity effect *F*-tests (*df* = 1, 25.7 to 77.8) and *t*-tests (*df* = 52.4 to 77.7) testing changes from Play to Still-Face episode 1 and 2.

	Overall effect		P vs. SF1		P vs. SF2		Activity	
	*F*	*p*	*t*	*p*	*t*	*p*	*F*	*p*
Test 1 (*n* = 23)								
RSA (ms)	0.17	.844	−0.53	.599			0.73	.397
logRSA/V_T_c (log ms/mL)	9.78	.001	−3.37	.001			0.42	.518
T_TOT_ (s)	1.10	.341	1.46	.149			0.91	.345
V_T_ (mL)	6.36	.004	1.32	.193			1.23	.273
HR (b/min)	8.67	.001	0.93	.359			14.18	.001
Test 1 and 2 (*n* = 16)								
RSA (ms)	1.46	.226	−0.25	.803	−1.59	.118	4.28	.043
logRSA/V_T_c (log ms/mL)	6.93	.001	−2.67	.009	−5.01	.001	0.23	.882
T_TOT_ (s)	5.56	.001	1.60	.115	3.97	.001	1.80	.184
V_T_ (mL)	5.06	.001	1.42	.162	3.05	.003	1.21	.276
HR (b/min)	3.83	.007	1.03	.309	2.06	.044	7.76	.007

*Note:* V_T_ = tidal volume; RSA = respiratory sinus arrhythmia; T_TOT_ = total respiratory cycle time; HR = heart rate; RSA/V_T_ = RSA normalized by V_T_; c = adjusted.

for total respiratory cycle time; logRSA = logarithm(RSA+1); logRSA/V_T_ = logarithm(RSA/V_T_)+1.

## Discussion

In this study we examined the role of respiration in the assessment and interpretation of RSA amplitude in 6-month-old infants. We found that respiration was significantly associated with RSA in at least one third of our infants and vagal withdrawal during a psychosocially challenging laboratory situation was demonstrated more consistently when controlling for respiration. However, RSA was not always readily detected in these infants, with 12.6% (range 0 to 58.2%) of breaths not allowing extraction of RSA because individual breaths did not accommodate more than one full cardiac IBI [5], [41], [53]. The fact that in some infants and episodes up to 58.2% of the breaths were too fast raises questions regarding studies that do not consider the specific respiratory dynamics of this age group. Because of the violation of the Nyquist rate criterion, such recordings may be subject to cardiac aliasing and thus erroneous extraction of artificially produced lower frequency components. Both gradual slowing of f_R_ [54], [55] and increasing coherence between high frequency heart rate variability and f_R_ [70] are observed with increasing age and are viewed as characteristics of respiratory system maturation.

Our breath-by-breath time-domain approach to RSA analysis readily identified instances of excessively fast f_R_ and selectively excluded them from the overall RSA analysis. This method can also quantify the extent to which a valid peak-valley RSA is detected in breaths that are long enough to allow RSA quantification. Across episodes and infants in these analyses, only 41% of all breaths that were sufficiently long for RSA extraction showed a valid peak-valley RSA. This lack of a continuous IBI time series would exacerbate the difficulties in applying frequency-domain methods to detect HR variability in the high frequency band. Studies of HR variability frequency components in neonates have often failed to identify substantial HR modulation in the respiration-related frequency range [53], [71] and RSA amplitude has been shown to increase throughout the first year of life [31], which could be due to an increasingly greater number of breathing cycles showing HR modulation compatible with a valid RSA. However, the origin of zero-RSA cycles is largely unexplored and both physiological explanations (lack of consistent vagal excitation, lack of respiratory system maturation, unreliable functioning of the respiratory gate) and methodological factors (interference of low-frequency fluctuations in the cardiac IBI time series, undetected artifacts or sensor problems in respiratory inductance plethysmography recordings) could account for this phenomenon.

We also found modulation of RSA by characteristics of the respiratory pattern, f_R_ and V_T_, in our infant sample. Although in adults, influences of both f_R_ and V_T_ on HR modulation have been demonstrated with considerable consistency [5], [9], [22], [23], [25], until now this phenomenon has only been explored in passing among infants [53], [70], [72]. Earlier research reported observations of a “breath-amplitude RSA” in infants with increases in RSA amplitude due to occasional slow deep breaths [73], [74]. However, the extent to which V_T_ impacts RSA on a breath-by-breath basis under conditions of variable behavioral challenge had not been systematically studied. Herein, we explored the role of V_T_ in modulating HR in infants. After controlling for f_R_ effects, we observed additional increases of RSA with higher V_T_ values, although these modulations were substantially smaller than those observed by T_TOT_ and were only found in approximately 20% of the infants. Notably, we used naturally occurring variations of both parameters, f_R_ and V_T_, across our experimental protocol, which may not allow direct comparisons with the pronounced respiratory RSA modulations observed with adults who perform paced breathing exercises across a wider range of breathing frequencies [4], [5], [7], [25], [75].

We also observed considerable between-individual variability in modulation of RSA by the respiratory pattern in these infants. Significant associations of f_R_ or V_T_ with RSA were detected in approximately one-third to one-half of infants, although most infants showed significant or marginal associations of RSA with at least one of the respiratory parameters. At an early stage of development, the extent of modulation may be linked to the integrity or maturation of the respiratory gating mechanism that has been proposed for RSA [1]. However, research with adults has also shown substantial variability in the extent of this modulation [25], and preliminary findings show associations of the magnitude of variability with disease-relevant aspects of asthma [26]. One functional interpretation of RSA is that it optimizes gas exchange [5], [76], [77]. Within this framework, a stronger modulation of RSA by respiratory pattern changes could provide a broader functional range for adjustment to changing environmental conditions and thus convey long-term health benefits [5].

Vagal withdrawal during the still-face challenge was readily shown when variance in RSA due to f_R_ and V_T_ was taken into account by our correction procedures. Our findings suggest that a correction for respiratory pattern effects is indicated when RSA is to be interpreted with respect to underlying vagal activity, echoing earlier work with adults [5], [9], [22]–[26]. The findings with corrected RSA indices confirmed the expectation of systematic decreases in cardiac vagal activity during the stressor episodes of the SFP. V_T_ and T_TOT_ increased significantly during Still-face episodes and both of these aspects of the respiratory pattern have been known from research with adults to increase RSA without necessarily changing vagal activity [5]. Controlling for these aspects by calculating the transfer function of RSA per ml V_T_ [9], [23], [25] and residualizing this index within the individual infant for T_TOT_ resulted in a more sensitive index of vagal activity. Notably, respiration-uncorrected RSA amplitude did not show significant changes related to the SFP administration in some of the analyses. Significant modulation was only observed when all five episodes were analyzed using all available breaths for the subsample of infants who completed all episodes. Thus, the traditional measure of RSA, which is equivalent to the high frequency heart rate variability, showed a limited sensitivity for demonstrating the expected cardiac vagal withdrawal under challenge. Research using respiration-uncorrected RSA in experimental emotional or cognitive challenges has remained equivocal, with studies showing no changes [48], decreases [43], [45], or increases [47] in infants. Studies have also shown considerable variability of uncorrected RSA indices during such challenges [42], [46], [49]. It should be noted that log transformation alone also improved the sensitivity of RSA, as log transformation improves the distributional characteristics of infant RSA, which shows a high number of respiratory cycles (59% in this sample) with zero-modulation of HR. However, findings were strongest with additional respiration-correction.

Of note, the significance of corrected RSA effects was retained after controlling for physical activity across episodes, indicating that the observed vagal withdrawal was due to factors related to the stressful experience rather than increased physical activity during still-face episodes. Most prior studies of psychosocial challenges failed to control adequately for physical activity effects (e.g., [6], [42]–[46]). The lack of association with physical activity may also be due to the rather narrow sample of behavioral states and activities within this laboratory protocol, as compared to ambulatory monitoring studies with adults, which have found a strong association between respiration-controlled RSA and physical activity levels [50]. However, HR was significantly associated with physical activity in all analyses. Alternatively, effects of physical activity may have been eliminated in the respiration-corrected RSA index because of the shared variance of respiration with activity. Thus, respiration-correction may also control for physical activity effects and therefore result in an index specifically related to emotional aspects of vagal withdrawal, whereas the respiration-uncorrected index showed some significant association with physical activity (significant for the subsample that completed both challenges).

The progressive decline in RSA values across the protocol for those infants who completed both tests may be of clinical interest. Longer-lasting reductions in cardiac vagal outflow due to repeated bouts of stress may impact the infant's ability to recover fully and thus could lead to long-term adjustments in cholinergic organ regulation. Longitudinal research is needed with larger samples assessed over various developmental stages to advance our knowledge of mechanisms and function of systematic changes in respiratory modulations of cardiac vagal outflow across developmental trajectories and their potential association with adjustment to environmental challenges and developmental outcomes.

Our study was limited by the small number of infants monitored and the reduction in sample size due to significant distress after the first Still-Face episode. We cannot rule out that results with the uncorrected RSA index may become more significant with a larger sample size; however, the lower sensitivity to the experimental effects compared to respiration-corrected RSA would be expected to remain. In addition, despite visible improvement in prediction of vagal withdrawal (from 7.1% explained variance with the uncorrected RSA index to 37.9% with the corrected index in the case of the ANOVA design of the first still-face exposure), the difference between these proportions of variance was not significant due to the small sample size. Thus, our findings suggest improvement in the right direction, but at this point cannot be substantiated further. Furthermore, our measure of physical activity was observational because accelerometery was not available in this infant set-up of the ambulatory respiratory inductance plethysmography device. However, given the high interrater-reliability of the observational measure, we felt sufficiently comfortable to interpret it. A limitation of our psychophysiology approach was that, in studying the association between respiratory regulation and vagal outflow, we had to rely on indicators more distal to these phenomena. Because RSA and respiratory pattern indices are derived from whole organ system behavior, they incorporate additional sources of variance that may obfuscate part of the association of interest. Measurements of motor nerve activity to the respiratory muscles and efferent cardiac vagal outflow would have been ideal but were obviously not feasible in a study of human infants with a focus on emotional responding. Finally, our strategy of correcting for respiratory factors deviated from those used for adults, which typically involves baseline calibrations for respiratory RSA modulation using a range of paced breathing frequencies [26], [75]. For obvious reasons, paced breathing is not possible in infants, but our approach was similar in that it was performed within-individuals and also involved the use of the transfer function [RSA/V_T_]; [ 9], [23,25]. Despite these limitations, we confirmed our main expectation that correcting for both f_R_ and V_T_ improved the sensitivity of this RSA parameter in demonstrating stress-induced vagal withdrawal.

*Conclusion.* The RSA amplitude varies systematically with f_R_ and V_T_ in 6-month-old infants. Respiration-controlled RSA indices were superior to non-corrected indices in demonstrating a predicted pattern of vagal withdrawal during challenging social interaction. Future research should implement one of the simple within-individual correction procedures of RSA for respiratory pattern aspects as tested in this study. Overall, a sizeable portion of breaths in infants is too short to provide the minimum amount of two IBIs for an extraction of RSA. This would indicate that caution is necessary to account for instances that do not provide the necessary preconditions for RSA extraction. Approaches such as our breath-by-breath time domain are necessary to avoid a loss in precision in RSA extraction and were shown to identify enough valid breaths for demonstrating the expected stress-induced vagal withdrawal. Enhanced precision in the measurement of the stress-elicited infant autonomic response promises to advance efforts to elucidate associations between early life physiological programming, psychosocial stress, and lung growth, development, and pathophysiology [78]–[81]. Future research needs to study the validity of respiration-correction procedures such as the ones examined here across the age span to enable a valid exploration of stress effects on the vagal system under a developmental perspective. This may eventually inform the development of a gold-standard for dealing with breathing related effects on RSA across ages.
